# The Treatment of Bronchitis

**Published:** 1893-03-04

**Authors:** 


### THE HOSPITAL FOR CONSUMPTION, BROMPTON.

### The Treatment of Bronchitis.

The varieties of bronchitis may be differently grouped according to the fancy of the writer. In this article on the treatment of bronchial inflammations at the Hospital for Consumption, the following classification will be adopted: 1. Acute bronchitis. This may be further sub-divided into (*a*) Bronchitis of the large and medium-sized tubes; (*b*) Capillary bronchitis. 2. Chronic bronchitis. 3. Plastic bronchitis. The third variety of the disease occurs so rarely, and its treatment is still so unsatisfactory, that it will hardly require further consideration.

The treatment of acute bronchitis will be considered as a whole, and special reference will be made where necessary to the procedure to be adopted in the case of capillary bronchitis.

At the outset it is of very great importance to determine whether the disease is primary or whether it is secondary to some organic lesion, or dependent on some other constitutional condition such as gout, albuminuria, &c.

In any event the patient must be kept in bed in a well-ventilated ward, the temperature of which should be maintained at a level of from 63 deg. to 67 deg. Fahr. The air of the room for the first few days should be kept moistened by means of steam from a steam kettle placed near the bed. The patient must be carefully protected from draughts, and while the steam is being used it must be remembered that there is danger of chill if the temperature of the room is allowed to fall. The action of the skin should be encouraged by means of warm drinks and the administration of diaphoretics. If necessary, a hot air bath may be employed for this purpose. The food should at first consist almost entirely of liquids, and the amount should be regulated according to the requirements of the patient. It should be taken at regular intervals, and care must be taken not to give too large quantities at a time.

The chest may be enveloped in a jacket poultice, made of linseed, or mustard and linseed, and changed every three hours. In weakly subjects poultices are better avoided. In most cases of bronchitis warm turpentine stupes applied alternately to the front and back of the chest give great relief to the pain and cough, and, as a rule, are to be preferred to poultices, which tend to embarrass the free movements of the chest. In cases of capillary bronchitis more energetic local measures are required. Vigorous counter irritation over the parts of the lung affected must be employed, and in some cases dry or wet cupping between the shoulders frequently gives much relief.

Venesection is rarely if ever called for, and should not be resorted to until all other measures have failed. Extreme engorgement of the right side of the heart and general lividity would be the indications for its employment.

It is usual to commence the medicinal treatment by the administration of a purge, consisting of calomel, with colocynth and hyoscyamus, or pulr. jalap co., followed on the next day by a saline laxative.

During the first stage of bronchitis a mixture, such as the following, may be given every four or six hours: ℞. ammon. carb., gr. iv.; vin. antimon., ♏ x.; vin. ipecac., ♏ x.; liq. ammon, acet., ʒss.; sp. æth. nit. ʒss.; with aq. chlorof., ℥j. Should the patient suffer from attacks of paroxysmal dyspnœa, due to bronchial spasm, the addition of a few grains (v.-viii.) of iodide of potassium to the mixture will usually afford immediate relief. Alkalies are also of much service during this stage of the disorder.

Sleeplessness may be treated by means of bromide of ammonium (gr. x.-xxx.), to which may be added chloral (gr. v.-xv.), provided the heart's action is powerful. The use of opium in cases of acute bronchitis must be entirely avoided. Signs of cardiac failure must be met by the employment of stimulants in the form of brandy. The amount prescribed must be regulated by the urgency of the symptoms.

In cases of capillary bronchitis free stimulation is generally required from the outset of the illness.

In addition to the brandy, a mixture containing carbonate of ammonia, gr. v., ether, ʒss.-ʒj., and liquor strychninæ, ♏ ii.-v., may be given every four hours. Should signs of cardiac failure manifest themselves, digitalis may be substituted for the liq. strychninæ, or added to the above mixture.

The inhalation of oxygen sometimes gives temporary relief in cases of capillary bronchitis.

When the secretion becomes free and copious the prescription first given must be changed for a more stimulating mixture. A combination of the tinct. of squills, ♏ x.—♏ xx., with ammon. carb., gr. v., and tinct. nucis vom. ♏ x., made up with aq. cholorof., ℥j., or inf. serpentaniæ, ℥j., or inf. senegæ, ℥j., will be found most useful. The local applications may now be discontinued, and the chest, which should be protected with cotton wool, may be rubbed night and morning with lin. terebin. acet.

Should the secretion become so profuse that the patient is unable to expectorate it, and there is danger of suffocation, an emetic must be administered.

A mixture of vin ipecac. (ʒss) with ammon. carb. (gr. xxx.), is the most useful combination to employ. The use of apomorphine is frequently followed by dangerous depression in these cases. Tickling the fauces or the administration of sulphate of zinc (gr. xv.-xxx.) may also be tried.

When the acute symptoms have subsided and the expectoration has diminished, it is advisable to give iron, combined with quinine and a vegetable bitter.

The irritability of the bronchial mucous membrane, should it exist, may now be alloyed by means of a linctus consisting of tr. scillæ ♏ x., tr. camph. co. ʒss, and syrup of tolu ʒj, to be taken when required.

In those cases of bronchitis secondary to heart disease, the employment of digitalis is frequently followed by the best results. Bronchitis connected with gout, alcoholism, &c., must be treated mainly with regard to the primary disease.

The treatment of the primary affection is generally followed by a marked amelioration of the pulmonary condition.

Should the bronchitis attack depend on the inhalation of some irritant, the patient should if possible be removed from the exciting cause of the affection.

*The Treatment of Chronic Bronchitis.*—The patient should avoid, as far as possible, all exposure to cold and damp. He should be warmly clad, and the general health should be improved by regular habits and good food. Should no constitutional affection underlie the complaint, the patient should be treated by means of tonics, such as iron, combined with quinine and an acid, and the administration of cod liver oil. The employment of the latter remedy is often followed by surprisingly good results. In those cases in which the bronchial cartarrh is the result of some employment, this must, if possible, be given up, and work of a more healthy description obtained.

If the pulmonary disorder is complicated by disease of the heart, rest, and the employment of cardiac tonics will improve the condition. Should gout be present, the exhibition of alkalies or colchicum will prove of service. In these cases, too, the administration of an occasional blue pill, followed by a saline, is often productive of great good. In the event of no cause being found for the pulmonary complaint, the tonic measures above mentioned must be persevered with, and symptoms must be treated as they arise.

Counter irritation is frequently of much service. For this purpose the chest may be rubbed night and morning with stimulating liniments, such as the lin. tereb. acet. Blisters may be used, or the chest may be painted with some preparation of iodine.

In those cases in which the cough is irritating and the expectoration slight in amount, relief may be got by the use of small doses of opium, combined with squills and syrup of tolu. The troch. glycyrrh. of the Brompton Hospital pharmacopœia, consisting mainly of ext. glycyrrh : ol : anisi : pulv. acaciae : and sugar often give excellent results. Should expectorants be given, the more stimulating kinds should be chosen. Carbonate of ammonia, combined with the tincture of nux vomica and the syrup of squills, or infusion of senega, give the best results. The tincture of digitalis may often be added with advantage. With profuse expectoration, the compound tincture of benzoin, with syrup of tolu and vinegar of squill, may be tried. Ammoniacum, cubebs, and especially tar, in the form of pills, and turpentine are all occasionally useful. The inhalation of steam alone, or medicated by the addition of tinct. benzoin co., terebine, or creosote, is sometimes productive of good results.

The bronchial catarrh is often complicated with severe paroxysmal attacks of dyspnœa. In such cases the hospital mixture under the title of mist. pot. iod. c. stramonio is of much service in relieving the bronchial spasm. This mixture has the following composition: R. Ext. stramonii, gr. ¼; ext. glycyrrhizæ, gr. i.; pot. iodidi, gr. iii.; ætheris chlorici ♏ v.; aquam ad ℥j. The dose of the iodide of potassium may be gradually increased to ten or even twenty grains. A combination of ether and tincture of lobelia with a few grains of carbonate of ammonia is also useful in these cases. The addition of strychnine or digitalis to either of these mixtures is sometimes of considerable service.

The paroxysm may be temporarily relieved by the inhalation of the fumes from a burning powder, composed of antispasmodic remedies. The pulv. stramon so., of the Brompton Hospital pharmacopœia, is much used for this purpose. This powder consists of pulv. stramon, ʒiv.; pulv. anisi, ʒjj; pulv. pot. nitrat., ʒii; pulv. tabaci, gr. v. A tea-spoonful may be placed on a plate, lighted, and the fumes inhaled. It is, however, very necessary that this remedy—and the caution applies to all preparations of a similar character—should be used with great care, especially in those cases in which there is cardiac weakness. Repeated and continued use of such powders may be followed by dilatation of the heart.

The embarrassment of the right heart, accompanied by venous engorgement, which at some time or other usually complicates chronic bronchitis, should be treated by free purgation, and the use of leeches over the cardiac area. In some cases venesection, followed by the administration of digitalis and other heart tonics, is productive of great and lasting relief. Change of air, especially to a mild, dry climate, is to be recommended, but, unfortunately, the advice can rarely be followed. Some patients derive considerable benefit from the use of a respirator.

